# Multifaceted regulation of thymic tolerance by pattern recognition cascades

**DOI:** 10.3389/fimmu.2026.1814309

**Published:** 2026-06-09

**Authors:** Soumyadeep Mukherjee, Paramita Paul, Arpita Kar, Subhasis Barik

**Affiliations:** 1 Department of In Vitro Carcinogenesis and Cellular Chemotherapy, Chittaranjan National Cancer Institute, Kolkata, India; 2 Department of Signal Transduction and Biogenic Amines, Chittaranjan National Cancer Institute, Kolkata, India

**Keywords:** autoimmunity, central tolerance, inflammation, pattern recognition, thymic selection, thymus

## Abstract

During their development, progenitor T cells have to pass through a series of tolerogenic filters inside the thymus, which ensure the survival and developmental advancement of only those thymocytes which fruitfully recognize the host’s own MHCs conjugated with non-self antigenic peptides. A set of microbial and host-derived biomolecules, called ‘patterns’, dynamically regulates the proficiency of this thymopoietic axis by a combination of thymocyte-extrinsic antigen presentation and thymocyte-intrinsic TCR signalling. Although well-characterized in terms of their impact on peripheral T cell tolerance, there is a lack of clarity regarding the influence of these patterns on the thymic tolerogenic checkpoints. From a clinical angle, this stands as a formidable weak point for the widely used immunosuppressive therapies against autoimmunity, which generically target these pattern recognition cascades. This review explores different aspects of the pattern recognition receptor-mediated regulation of key thymic events from an exclusively tolerogenic perspective, and unravels the mechanistic complexity underlying their impact on thymic tolerance. As the pattern recognition-mediated signalling cascades constitute a significant branch of the inflammatory network, inferences from this review elaborate a frequently overlooked collaboration between inflammation and self-tolerance; highlighting the need for potential therapeutic repurposing against autoimmune diseases.

## 1 Introduction

The ability to distinguish non-self from self is a defining characteristic of the adaptive immune system. This self-tolerance is imparted to the T cells at two stages: by eliminating potential autoreactive clones centrally during their intrathymic development and later, by limiting their activity at the periphery through various restrictive mechanisms (1). Breach of these tolerogenic modalities results in aberrant, self-directed immune hyperactivity, known as autoimmunity; which presents itself as an unimpeded, auto-amplifying inflammatory response (2, 3). Among different signals seeding a cascade of such reactions, the ones with defined molecular patterns come as front-liners. A specific set of receptors, a.k.a. pattern recognition receptors (PRR), are designated for recognising the patterns and mounting an inflammatory response under different contexts (4). Activation of these pattern-induced signalling pathways and the downstream inflammatory outburst are indispensably tied to the clinical manifestations of multiple autoimmune diseases (5–7). Despite the plentitude of cutting-edge treatment protocols against autoimmunity (8, 9), immunosuppressive therapies, principally targeting these pattern-regulated inflammatory checkpoints, have been the most preferred approach for such diseases (10). However, irrespective of its unequivocally regarded efficacy in restoring immune tolerance at the periphery, how immunosuppression specifically modulates central tolerance has remained elusive.

Progenitor T cells pass through a winding developmental path to acquire their antigenic responsiveness and immune competence (11, 12). A major part of this development occurs within the thymus, where precursor T cells, a.k.a. thymocytes are educated about the self-antigenic repertoire (13, 14). This educational programme is extremely important in enforcing thymic tolerance: the state where the thymus maintains immune alloreactivity by imposing certain constraints to the developing thymocytes. As antigen processing and presentation are integral parts of a tolerogenic process, multiple functional aspects of thymic tolerance require a basally active innate immune response (15). Generic immunosuppression may thus even harm thymic tolerance and foster autoimmunity (16–18). This necessitates an even more meticulous understanding of the complex crosstalk between inflammation and autoimmunity in the context of central tolerance.

Besides being one of the earliest triggers for a full-fledged autoimmune inflammation, pattern recognition signalling acts as a dynamic regulator of thymic function. This review hence revisits the existing findings regarding the pattern-induced thymic as well as thymopoietic manifestations from a tolerogenic viewpoint, which has thus far remained less explored. Contrary to the generalized notion of their antagonistic impact over immune tolerance, conclusions from this review highlight the beneficial contributions of the PRR signalling modules towards thymic tolerance. Given the intricate involvement of inflammatory signals in the maintenance of thymic tolerance, systematic understanding of the implications of these inflammatory mediators on the thymic tolerogenic modalities might facilitate the repurposing of prototypical immunosuppressive therapies, which may help manage autoimmunity at its origin.

## 2 Thymic tolerance: the central barrier to autoimmunity

**Figure 1:**
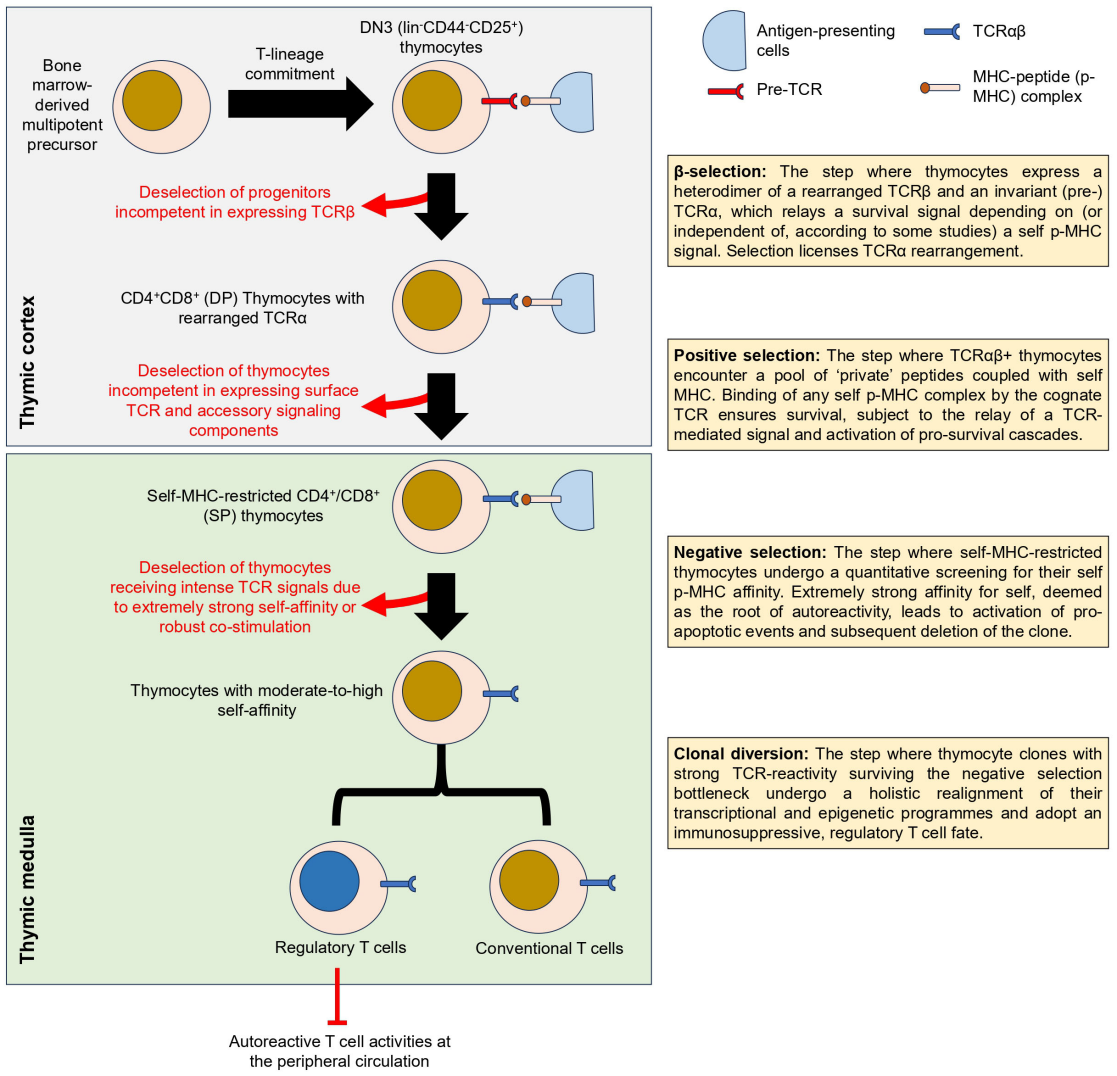
A snapshot of the intrathymic tolerogenic landscape. Establishment of tolerance within the thymus is a multi-step process that begins as early as the entry of bone marrow-derived hematopoietic progenitors into the thymus, through a combination of T cell receptor-mediated signal intensity and a reprogramming of their transcriptomic profile. In the initial, pre-tolerogenic phase, instructive signals such as Notch, IL-7 etc. guide the multipotent early progenitors to T-lineage commitment. The earliest T-lineage-bound progenitors a.k.a. DN3 thymocytes express a premature form of the T cell receptor, also called pre-TCR, which relays a signal depending (or independent of according to others) on peptide:MHC molecules presented by the cortical thymic stromal cells. Only those DN3 thymocytes which are able to successfully transduce this signal are allowed to survive and rearrange their TCRα locus. Following the expression of a fully rearranged TCRαβ heterodimer, CD4+CD8+ thymocytes are screened for their ability to ligate their TCR to any of the host’s self MHC in conjunction with a set of thymic cortex-specific private peptides. Thymocytes failing this are eliminated by various programmed death modules, while the successful ones traverse to the medulla while retaining the expression of any one between CD4 and CD8 (based on their MHC restriction). These single-positive thymocytes undergo a quantitative peptide:MHC binding in the medullary stroma. Strong binding to the ubiquitous self peptides coupled with the self MHCs in absence of sufficient co-stimulatory signals are considered as the gateway to autoimmunity, and such potential autoreactive thymocytes are removed by a process termed ‘clonal deletion’. This process forms the basis of the negative selection process, which keeps the thymus away from producing autoreactive T cells. On the other hand, strong TCR signals supplemented with pro-survival cues such as IL-2, and a holistic reprogramming of the transcriptional circuit towards FoxP3-dependence redirects a thymocyte towards a regulatory T cell fate. Such thymus-derived regulatory T cells play an important part in mitigating intrathymic and extrathymic autoimmunity by their wide range of immunosuppressive mechanisms.

The major goal of the T cell developmental proceedings is to confer the T cell precursors with T cell-identity and more precisely, T cell receptor (TCR)-responsiveness, i.e. the ability to recognise peptide:MHC (pMHC) complexes displayed by the antigen-presenting cells (APC) and mount a response. However, before a T cell precursor is fully developed and allowed to be released into the system, they need proper schooling about the host’s antigens so that they do not recognise those as potential threats. The thymus achieves this by putting all developing thymocytes under a fastidious screening regimen that selects thymocyte clones with just-the-right TCR affinities for the self-pMHC complexes (19). This entire process, termed thymic selection, consists of a chain of events that keeps on eliminating thymocytes with non-optimal TCR signal strength or rerouting them towards alternative developmental fates (**Figure 1**); leading to a directed evolution of the randomized TCR repertoire towards alloreactivity (20).

### 2.1 Setting the stage for tolerogenic selection

After a stipulated dose of Notch signalling followed by T-lineage commitment, the bone marrow-originated CD4-CD8- (double-negative, DN) intrathymic progenitors start expressing an immature form of the TCR heterodimer. This precursor TCR, also called the pre-TCR, consists of a rearranged TCRβ and an invariant TCRα, providing the progenitors with a signal that enables them to survive, proliferate and initiate rearrangement of the TCRα locus (21). Thymocytes with out-of-frame TCRβ rearrangements, or rearrangements with in-frame premature stop codons are filtered out at this stage. Despite disputes regarding the dependence of pre-TCR on thymic self pMHCs, recent reports suggest this TCRβ-dependent ‘β-selection’ event to be the first developmental checkpoint that establishes T-lineage identity in the precursors (22) and dictates the extent of TCR junctional diversity in the T cells, which grossly governs their autoreactive features (23). Following β-selection, the CD4+CD8+ (double-positive, DP) thymocytes, equipped with a fully rearranged TCRαβ, are put to test to check if they recognise any self MHC at all. This is particularly important as MHC molecules are polymorphic in nature, and no structural element in the TCR can guarantee its binding to the MHCs of all possible haplotypes. Thymocytes bearing TCRs that can bind to any of the self pMHCs of the individual host are ‘positively selected’ and upregulate CCR4 and CCR7 to reach the CCL17, CCL19 and CCL21-rich thymic medulla (19). Thymocytes failing this test because of incompetent TCRs have two options: either to edit their TCR reading frame (‘processive rearrangement’) to increase the stochasticity of self pMHC binding and survival (24), or to stay incompetent and succumb to ‘death by neglect’ (25). Although positive selection lacks any apparent connection with tolerance, failure to establish unresponsiveness to low-affinity antigens leads to systemic autoimmunity.

### 2.2 Execution of thymic tolerance

Auditioning for potential self-reactivity begins in the medulla, in a process termed ‘negative selection’ (**Figure 1**). All positively selected thymocytes (expressing either CD4 or CD8 based on their MHC restriction), including the autoreactive ones, are again allowed to bind self-pMHC complexes on the medullary APCs. TCR-MHC interaction at the medulla, however, imposes an additional requirement for co-stimulation as well as IL-2 responsiveness. Thymocyte clones receiving a strong TCR signal, coupled with strong co-stimulation, die in a process called ‘clonal deletion’ (26). Moreover, when these strong TCR and co-stimulatory signals are supplemented with a pro-survival cue such as IL-2, the clones survive and are redirected (‘clonal diversion’) towards immunosuppressive regulatory T (Treg) cell fate by the induction of a FoxP3-dependent, Treg-specific transcriptional signature (27). A moderate TCR signal, on the other hand, keeps the thymocytes on track for conventional T cell development without further ado (20).

### 2.3 Antigen presentation dynamics in thymic selection

While the purpose of this selection programme is intended towards thymocytes, thymic APCs are indispensable in this process. These APCs, including the thymic epithelial cells (TEC), macrophages, dendritic cells (DC), B cells etc, are endowed with an extremely high capacity of proteolytically processing endogenous antigens and presenting them with self MHCs. Cortical TECs (cTEC) express a unique proteasomal subunit named β5t, which constitutes the thymoproteasome (28); along with several unique lysosomal proteases like cathepsin L, thymus-specific serine protease (TSSP) etc (29). This gives a peculiar substrate specificity to the proteolytic machinery within the cTECs, which can produce ‘private’ peptides: antigenic peptides exclusive to the cTECs. Thymocytes positively selected against such peptides are free from possibilities of re-encountering the same peptides elsewhere in future and initiate an autoimmune response (or re-encounter them in the thymic medulla and fall prey to clonal deletion). Medullary TECs (mTEC) as well as other medullary APCs, on the other hand, display a considerably ‘public’ repertoire of self peptides to emulate the peripheral antigenic profile and identify potential self-reactive clones. These public peptides are either ubiquitously expressed or of diverse tissue origin, which are derived from a set of tissue-restricted antigens (TRA) expressed randomly yet in an order within the mTECs (30). Specialized transcription factors such as autoimmune regulator (Aire), forebrain embryonic zinc finger 2 (Fezf2) etc control different aspects of mTEC function, including TRA expression (31). Medullary APCs, unlike their cortical counterparts, also express handsome amounts of co-stimulatory ligands due to their requisition for the enforcement of negative selection (29); and can cross-present antigens acquired from adjacent APCs, with stronger tolerizing effects (32).

## 3 The ‘patterns’ of inflammation

The discretion between self and non-self is seldom clear, and not dichotomous at all. Therefore, tolerance to the self and intolerance to the non-self is not advantageous for the host under different situations. Although the indigenous microflora is foreign to the host, the immune system needs to be tolerized to them in order to facilitate their sustenance (33). On the other hand, during a systemic viral infection, there is a high risk of viral epitopes being presented by thymic APCs and the thymocytes recognizing those as self to become tolerized; which is evidently detrimental for the host (34). Therefore, the host requires elegant tuning mechanisms to control the thymic tolerogenic process.

In aesthetic terms, ‘pattern’ means a universal, repetitive design. Biological patterns range from superficial elements (bacterial lipopolysaccharides or lipopeptides) to intracellular components (nucleic acids) of various microorganisms. Recognizing these microbe/pathogen-associated molecular patterns (MAMP/PAMP) and sending an ‘intruder alert’ to the system is beneficial for the hosts so as to promptly mount an innate immune reaction against the foreign bodies and grant the system enough time to generate a precise adaptive immune response (4), which is necessary for the build-up and long-term storage of immunological memory. Interestingly, certain host molecules called damage-associated molecular patterns (DAMP) also have the ability to act as patterns when present in inappropriate places due to excessive cell damage and lytic death (35). The conglomeration of these signalling networks decides a cell’s fate in multiple aspects, such as survival, proliferation, differentiation and global adaptability to stress.

The PRRs are named and categorized according to the first member identified among a homologous group of similar receptors. For example, Toll-like receptors (TLR) (36) and nucleotide-binding oligomerization domain (NOD)-like receptors (NLR) (37) respectively represent families of PRRs with evolutionarily conserved structural and mechanistic resemblances to *Drosophila* Toll and plant NOD proteins. Apart from them, three more major PRR clusters: Retinoic acid-inducible gene (RIG)-I like receptors (RLR) (38), Absent in melanoma (AIM)-2 like receptors (ALR) (39) and C-type lectin receptors (CLR) (40) are reported. Interestingly, PRRs belonging to different families and even within the same family have widely different ligand preferences. Cell surface-expressed PRRs (such as TLR2, TLR4, TLR5 etc.) detect extracellular patterns such as bacterial or viral surface components, while cell-intrinsic PRRs (such as TLR7, NLRP3, MDA5, cGAS etc.) have selective affinities for patterns which are usually cytosolic, including the pathogen-derived DNA and RNA (4). Such omnipresence of the PRRs allows uninterrupted survey and signal integration from the cell-exterior as well as interior, imitating an OR-gated circuit with multiple inputs.

The fundamental mechanism of signal transduction is fairly similar across different PRR subgroups. Ligand binding-induced dimerization of PRRs allows their leucine-rich repeats (LRR)-containing endodomains to interact with adaptor proteins (such as MyD88, TRAF6 etc.), which further relay the signal through a multi-nodal network to downstream transcription factors (such as NF-κB, NFIL-6 etc) (41). Notably, the transcriptional response varies from one PRR to the other, and often involves consistent cross-regulation among different PRRs. The eventual outcome depends on the extent of activation of each of these signalling branches as well as their cross-regulation (42), having widespread biological significance.

## 4 Pattern recognition and thymic tolerance: a boon in disguise?

PRR signalling is one of the preliminary signalling pathways driving the colonization of the foetal thymic rudiment by seeding progenitors (43). Incidentally, these same PRR signals (albeit at heightened levels) lead to thymic degeneration and the emergence of autoreactive T cell clones during aging and different inflammatory pathologies (44–46). While one may consider this as a simple instance of ‘antagonistic pleiotropy’ (47), the arguments of such contextual regulation being evolutionarily selected in favour of the integrity of central tolerance cannot be overlooked (48); especially for the complex mutualism between inflammation and autoimmunity at the periphery. Below we revisit the key paradigms associated with the interplay between the patterns and thymopoiesis: in light of their potential tolerogenic implications.

### 4.1 Striking the balance between load and capacity

The T lymphopoietic axis may be considered as a long pipeline; with multipotent progenitors entering from one end and mature CD4+ and CD8+ T cells exiting from the other. Along the length of this pipeline lies the thymic self and non-self pMHCs, awaiting recognition and binding by the TCRs from developing thymocytes. Going by the laws of probability, the flux of progenitors inside the thymus needs to be balanced in order to avoid dilution of these pMHCs for a thymocyte undergoing selection (49).

**Figure 2:**
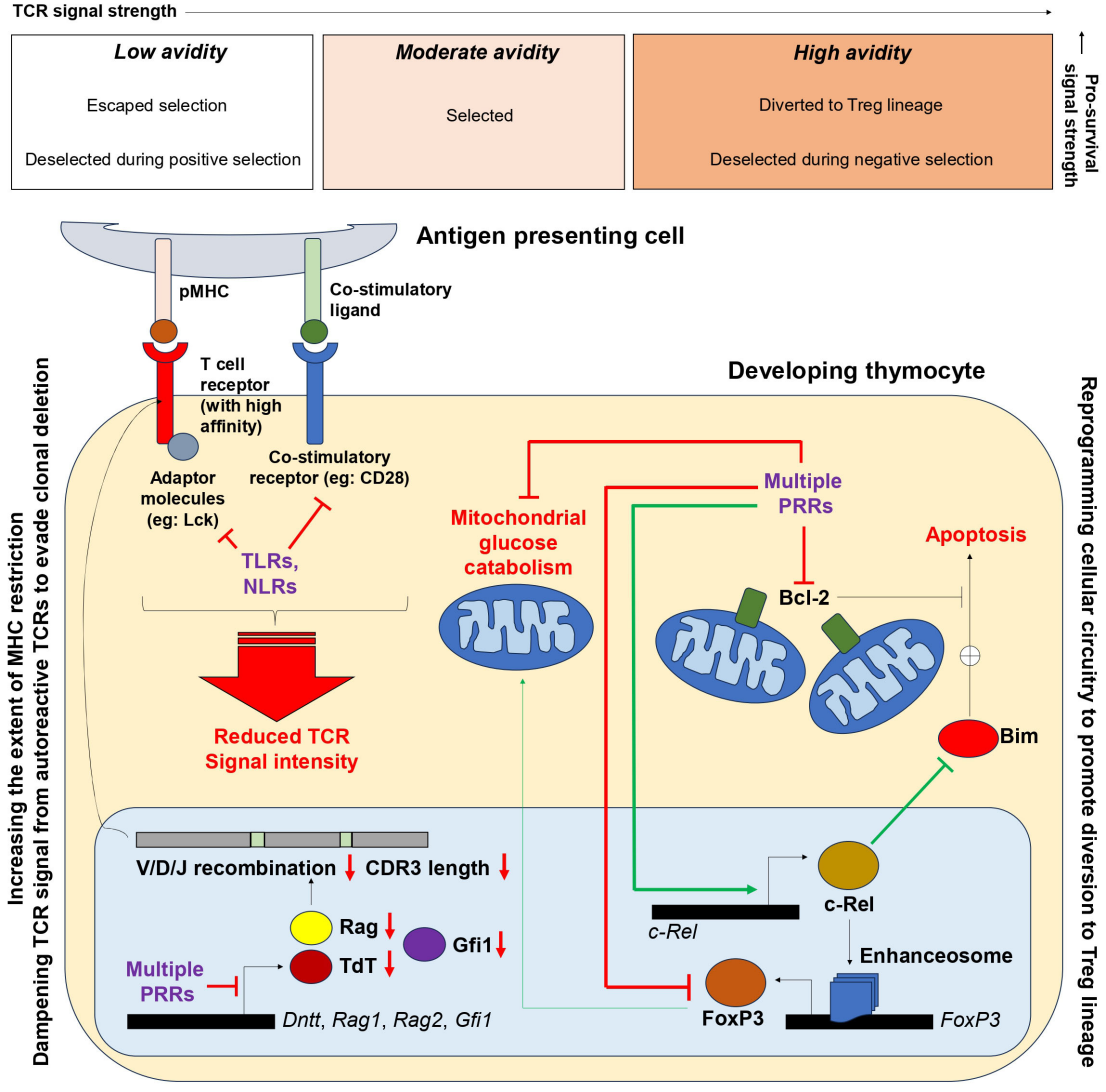
Pattern recognition-based control of thymocyte-intrinsic tolerogenic events. Thymic selection modalities impinge on thymocyte fate through two distinct yet interlinked ways: survival and Treg-diversion. Multiple branches of pattern recognition signaling control thymocyte survival in response to TCR-pMHC interaction via three non-overlapping modes. The first mode involves restricting the stereochemical features of the TCR CDR3 region to a high-intensity signaling competence. This is achieved by PRR-dependent downregulation of genes which encode proteins with active involvement in V(D)J recombination and N-nucleotide addition, allowing PRRs to enforce a regulation on CDR3 sequence diversity and physicochemical properties such as hydrophobicity. In parallel, PRR-mediated signals are key to controlling the assembly of the TCR signalosome and maintaining the balance between pro- and anti-apoptotic signals in response to TCR activation. These branches are cardinal to the restriction of the TCR signals to a moderate level that is conducive for thymocyte survival, and at the same time are the mechanisms which allow autoreactivity to creep in such clones. Several arms of these pattern-mediated cascades also directly promote or antagonize the establishment of a FoxP3-dependent Treg-signature. Activation of c-Rel, a NF-κB family transcription factor acting downstream of multiple pattern recognition cascades, is induced upon multiple pattern-dependent stimuli, which forms an enhanceosome complex with several other transcription factors upstream of the FoxP3 gene. This enhanceosome formation is an irreplaceable requirement for the transcriptional activation of FoxP3. TLR-mediated signals are, again, important in countering the proteostasis of FoxP3 and hindering the FoxP3-dependent metabolic rewiring required for the immunosuppressive functions of regulatory T cells.

The bone marrow (BM) hematopoietic stem and progenitor cells (HSPC) express a wide range of PRRs. Basal TLR: Myd88 signalling promotes the generation of lympho-myeloid progenitors and the maintenance of HSC quiescence in the mouse embryonic aorta-gonad-mesonephros (AGM) region (43). Maintenance of the adult HSPC pool depends on transposable repetitive element-dependent triggering of the RIG-I pathway as well (50). Systemic TLR activation, on the other hand, breaks the dormancy of quiescent HSCs (51) as well as the entire Lin-Sca1+Kit+ (LSK) population (52, 53); forcing them towards rapid proliferation in a TRIF-dependent, Myd88-independent manner. Even in absence of infections, extracellular ATP activates NLRP3 to promote HSPC mobilization by increased accumulation of CXCR4 and Sphingosine-1 phosphate receptor 1 (S1PR1) in their membrane lipid rafts (54). Chronic low-dose TLR stimulation, contrarily, depletes the HSC pool and lowers their repopulating potential (55). Expression of Ki-67, a cell proliferation marker, is gradually lost from all pre-thymic progenitors following a surge of upregulation during continuous application of TLR agonists (56), indicating a hormetic effect of the TLRs on progenitor proliferation. ZBP1 activation even triggers necroptosis in HSPCs (57). Speculatively, such regulation of HSPC proliferation and survival by PRRs may be accomplished in part by controlling the dynamics of Notch ligand expression on the BM stroma (58). The scenario within the thymus is also quite similar. While the majority of studies report significant annihilation of the T-lineage-committed (DN2, DN3) thymocytes upon PRR stimulation along with an accumulation of DN1/ETP (59, 60), one study (56) shows a drop in ETP frequency as well upon LPS and poly(I:C) treatment. Nevertheless, a universal explanation behind the PRR-induced clearance of the relatively mature T cell progenitors still remains obscure. TLR/RLR agonism usually renders the thymocytes (especially the relatively immature ones) more susceptible to apoptosis (45, 59). Despite canonical views regarding dampened pro-survival signalling as a downstream response to TLR agonists in thymocytes (44), recent reports suggest the presence of unique suppressive mechanisms to protect semi-mature thymocytes from the cusp of cGAS/STING-dependent apoptosis (61). Again, prior priming of TLR7 significantly reduces sepsis-induced thymocyte mortality (62), indicating that PRRs differentially modulate the apoptotic endurance of thymocytes based on the intensity and longevity of inflammation. The dynamics between PRR signalling and thymocyte apoptosis is highlighted in **Figure 2**.

**Figure 3:**
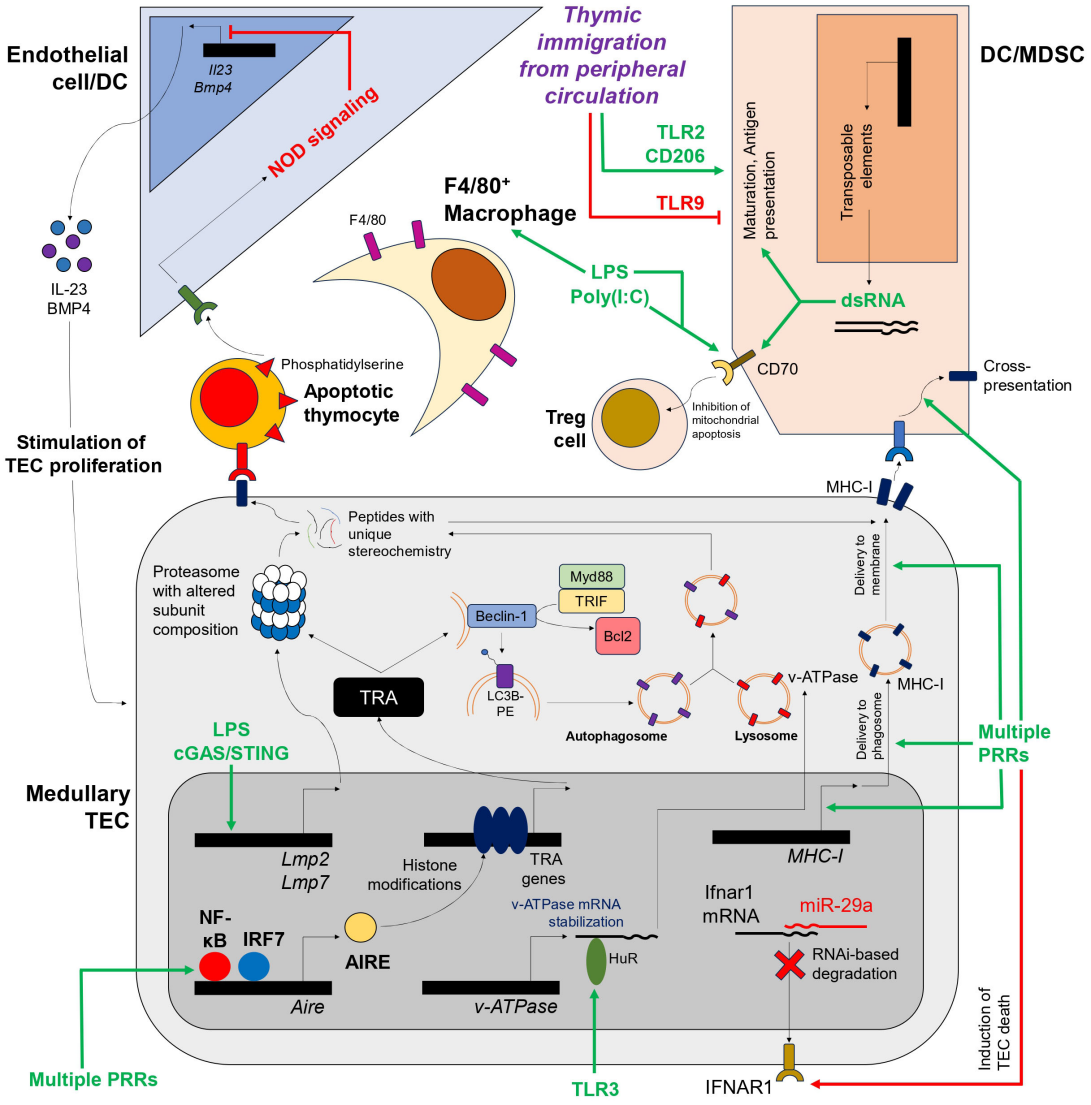
Influence of pattern recognition cascades on thymic antigen presentation. Pattern recognition-dependent signals control different facets of intrathymic antigen presentation. Within the medullary thymic epithelial cell (mTEC) compartment, different PRR-mediated cues tune the production (through Aire), processing (through the modulation of autophagic and proteasomal degradation dynamics) and presentation (through MHC expression kinetics) of self-antigens. Aire expression is under active control of transcription factors such as NF-κB, IRF7 etc. which are dynamically regulated by different pattern recognition receptors. Different patterns induce the expression of key immunoproteasome subunits Lmp2 and Lmp7, which alters the spectrum of proteolytic properties of these proteasomes. On the other hand, PRR cascades adaptors Myd88 and TRIF promote the initial stages of autophagosome formation, while v-ATPase mRNA stabilization acts as a potent mode of aggravating autophagosome acidification in response to pattern signals. The peptides produced by these modes, having unique stereochemical features, are associated with MHCs and presented on the cell surface in a process under active regulation by the PRRs. Culmination of these events are key to the contextual dynamics of intrathymic antigen presentation and their long-standing impact on thymocyte selection. Pattern-induced type I interferon signals are also involved in controlling the life/death decisions of TECs, thereby differentially regulating the productivity of the thymic tolerogenic pipeline during homeostatic and pathogenic conditions. Non-epithelial antigen-presenting cell-intrinsic pattern recognition signaling drives their thymic immigration and antigen cross-presentation. Endothelial/dendritic cell-intrinsic NOD signaling, triggered by apoptotic thymocyte detection, represses thymic regenerative factors; bridging pathogenic inflammation to thymic selection and thymic health.

Thus, patterns are key to the generation and sustenance of the pre-thymic progenitors and their recruitment to the thymus, along with the controlled depletion of developing thymocytes under pathologic settings. This puts the thymus under tremendous pressure to pass as many progenitors as possible through the selection filters in a rush; requiring an increase in the thymic APC pool. PRRs tackle this task in a context-specific manner. Tonic TLR signalling in the thymic epithelium is strongly tied to the development of medullary thymic epithelial cells (mTEC). The *Aire* ORF contains a highly conserved 90-nucleotides-long NF-κB responsive enhancer ~3 kb upstream of the coding region (63), whose functionality obligatorily requires the alternative NF-κB subunit RelB (64). Furthermore, IRF7, another PRR-activated transcription factor, is essential for the establishment of *Aire* expression in the developing mTECs (65). ScRNA-seq of the human thymus concordantly reveals AIRE+ mTECs to be rich in several TLR transcripts (66). TRAF3, an inhibitor of non-classical NF-κB signalling, poses a roadblock for AIRE^+^ mTEC development which can only be overcome upon their crosstalk with single-positive thymocytes as well as the RANKL-RANK pathway (67). Nevertheless, following suit, pathologic PRR signalling begets negative consequences for the TECs, whose cellularity drastically decreases in response to several patterns (46, 68). Such reduction in TEC levels concomitant with increased progenitor load may accelerate the collapse of central tolerance, which may hold significant importance in alleviating tolerance to infectious agents by enforcing molecular mimicry (69). Interestingly, high TLR4, TLR7 and TLR9 expression is observed in the thymi of individuals affected by autoimmune diseases such as myasthenia gravis, which show either thymic hyperplasia or involution (70, 71). From the host’s point of view, such overgrowth of thymic medullary tissue during autoimmune diseases might be considered a desperate (and often futile) attempt to restore the already-defective central tolerance; whereas involution might come off as the consequence of its proliferative exhaustion. The TEC-intrinsic noncoding RNA network, which has been found to set the threshold for the induction of pattern-induced, interferon-dependent death signalling in them (72), might be looked upon as a prospective regulator of thymic fate in response to pattern-mediated signalling. Of note, the dependence of thymic growth on thymic endothelial and dendritic cell-intrinsic NOD signalling, which post-transcriptionally represses several TEC growth factors (such as IL-23, BMP4 etc) by sensing the abundance of phosphatidylserine on thymocytes (73), might be accountable for the contextual dichotomy between thymic hyperplasia and involution in response to inflammation by bridging the cell-extrinsic signals to the intrinsic non-coding RNA network (briefly depicted in **Figure 3**).

### 4.2 Controlling the thymic ligandome

Rather than the absolute numbers of APCs, the extent of their antigen acquisition, processing and presentation capacity decides the tolerogenic efficiency of the thymus. PRRs dynamically modulate each step of this axis to suit the need of the system (**Figure 3**). Steady-state PRR signalling is a critical determinant of Aire expression in mTECs, which has a key role in de-repressing the silent TRA loci (74). On the other hand, phagocytic capacity of non-epithelial thymic APCs is dynamically modulated when stimulated by different PRR ligands (75, 76). Several PRR pathways play crucial roles in intracellular macromolecular turnover, leading to dynamic changes in the thymic APC-intrinsic degradome. Macroautophagy, one of the central pathways leading to self antigen digestion, is actively regulated by PRR signalling. Myd88 and TRIF physically associate with Beclin-1 and disrupt its interaction with Bcl-2, aggravating the induction of autophagy (77). Vacuolar acidification, a prerequisite for lysosomal maturation, is under direct control of TLR3, which stabilizes the mRNA of different v-ATPase subunit-encoding genes in a HuR-dependent manner (78). Different PAMPs like LPS, poly(I:C), dsDNA as well as synthetic TLR stimulants like imiquimod increase v-ATPase-dependent LC3B lipidation and its punctation on vesicular membrane, driving enhanced autophagosome-lysosome fusion (79, 80). NLRP3 inflammasome activation, interestingly, inhibits autophagy by caspase-1-mediated cleavage of TRIF and downregulation of autophagy-associated genes (81). LPS as well as the cGAS/STING-pathway have been found to upregulate the key immunoproteasomal subunits LMP2 and LMP7, inducing marked alterations in its trypsin-like and chymotrypsin-like activities (82, 83); which produce antigenic peptides with distinct stereochemical effects on the TCR:pMHC interactions and their strength (84).

Topical TLR7/8 activation decreases resident dendritic cells (DC) and increases migratory DCs in the thymus, without any significant change in the proportion of thymic plasmacytoid dendritic cells (pDC) (68). pDC-intrinsic TLR9 signalling, however, decreases their thymus homing by downregulation of CCR9, resulting in an impairment of peripheral antigen transport to the thymus and central tolerance (85). Contrarily, mannose receptor-mediated internalization of hepatitis B virus (HBV) surface antigen (HBsAg) by the monocytic myeloid-derived suppressor cells promotes their thymic deployment and medullary cross-presentation (86), acting as a significant branch of HBV-mediated immune tolerization (87). Again, genetic ablation of TLR2 in mice leads to reduced thymic migration of pDCs and shrinks the thymic PLZF+ unconventional T cell pool (88); highlighting differential effects of individual PRRs on thymic migration of antigen-loaded peripheral APCs. The thymic F4/80+ macrophage population is expanded in response to LPS: mainly during the tolerance phase (89), indicating an important role of these APCs in restoring thymic tolerance in an involuted thymus undergoing recovery. However, whether these macrophages actively participate in antigen presentation or only scavenge the deselected, apoptotic thymocytes (90), is not clear. Myd88-dependent TLR signalling in mTECs drives the emergence of a CD14+SIRPα+ monocyte-derived DC population which cross-presents mTEC-derived antigens (91). Beside increasing MHC-I transcription in the stromal cells (92, 93), multiple PRRs endorse the delivery of MHC-I from endosome to phagosome and promote antigen cross-presentation by DCs (94, 95). Furthermore, TLR ligands such as LPS, poly(I:C) etc increase CD70 expression on DCs (96), which is essential for the survival of thymic Treg cells at the positive selection step through the attenuation of mitochondria-dependent apoptosis (97). Incidentally, transposable element-derived dsRNA sensing and consequent activation of RIG-I/MDA5 are more active in thymic pDCs than their extrathymic counterparts, actively regulating their tolerogenic functions (98). Thus, the outcomes of distinct PRRs on intrathymic antigen presentation are widely varied and hold monumental tolerogenic implications (**Figure 3**).

### 4.3 Adjusting TCR signal intensity

While the TCR-pMHC interactions during T lymphopoiesis may appear completely stochastic, certain structural features in the TCRs govern their antigenic affinity. Each TCR is made of two subunits, each of which is expressed by a rearranged DNA sequence with three segments in tandem: variable (V), diversity (D) and joining (J) (25). Variability of TCR repertoires across individuals arises by combinatorial pairing among different TCR chains, which themselves are diverse among each other due to the presence of variable palindromic (P) or non-templated (N) nucleotides at the coding ends of individual V, D and J segments. These additional nucleotides, added by an enzyme named terminal deoxynucleotidyl transferase (TdT), usually span the region encoding the domain responsible for recognizing and interacting with the epitope on the pMHC. This region, called complementarity determining region (CDR) 3, naturally carries the majority of diversity across the entire TCR chain (99). Strikingly, the autoreactive T cells have been found to bear TCRs with abnormally short CDR3 sequences, along with an enrichment of hydrophobic amino acids in it (100); which stereochemically lowers the threshold for kinetic proofreading by the TCR/LAT signalosome (101). Besides, there is an increased sharing of TCR repertoires across individuals with similar autoimmune pathologies.

Competence to rearrange and express TCR transcripts requires prior priming of T-lineage-bound progenitors, even before their thymic entry. HSPC-intrinsic PRR signalling dampens these events by a global downregulation of T-lineage restriction-related genes (56). This attenuation of T-lineage signature comes with a significant myeloid or innate lymphoid lineage bias; offering the system an alternative route for emergency haematopoiesis in response to inflammatory cues (102, 103) and even for bolstering intrathymic negative selection (104). Analysis of the thymic transcriptome after intraperitoneal LPS challenge in C57BL/6 mice exhibits notable dysregulation of events related to T cell development. Surprisingly, several genes related to the early development and T-lineage commitment of thymic progenitors (including the master transcription factor *Bcl11b*) are upregulated, whereas expression of genes related to V(D)J recombination (*Rag1*, *Rag2*), β-selection (*Cd27*, *Cd28*) and TCR signalling (*Lat*, *Zap70*, *Lck* etc as well as CD3 and TCR subunits) shows a steep reduction (105). These findings strongly suggest that PRR activation dampens TCR locus rearrangement as well as TCR signal intensity, and not T-lineage-priming, in developing thymocytes. The compromised V(D)J recombination, evident by lower levels of TCR excision circles in the recent thymic emigrants in response to poly(I:C) (59), also corroborates the reduced levels and activities of RAG recombinases in them. Additionally, PRR stimuli downregulate TdT expression, resulting in the shortening of CDR3 sequences, as evident in the case of TLR7-transgenic mice with systemic lupus-like symptoms, whose T cells exhibit reduced TCR clonality, usage of unusual Vβ and Jβ segments and sharing of extremely high CDR homology across individuals (106). Though the attenuated TCR signalling in such potentially autoreactive thymocytes may result in insufficient clonal deletion, these very features of the TCR enhance MHC restriction (19), plausibly precipitating an increased yield of altered self-specific T cell clones during sustained infections (107). NOD1 and NOD2, two bacterial peptidoglycan sensors, have in fact been found to strengthen the positive selection of CD8+ thymocytes by modulating ERK phosphorylation (108). Notably, the ablation of inflammasome components NLRP3 and ASC delays the loss of TCR polyclonality with aging (46). Although CDR3 sequences return to normal in terms of length and hydrophobicity during recovery from thymic involution by the withdrawal of the PRR agonist (59) or during the development of tolerance to the specific PRR ligand (89), TCR signal strength remains significantly low. This implies the autoimmune consequences of PRR signalling on T lymphopoiesis to be reversible, at least against a non-chronic regimen of PRR agonism; albeit at the cost of a dampened TCR signalling capacity in the developing thymocytes. Of note, this suboptimal TCR signal receptivity is a signature of autoreactive T cells at the periphery too, which is generally attributed to PRR-dependent immunological feedback inhibitory mechanisms (109). Thus, developmental and post-developmental events collaborate in bringing down the TCR sensitivity in autoreactive T cell clones, with PRRs acting as multiphasic regulators of the process (**Figure 2**). This hyporesponsiveness might benefit the host by limiting their activation and consequent cytotoxicity in the periphery only in response to a sufficient level of inflammatory cues (110) as in the case of infections (which may even be relayed by the PRRs themselves (111)), but not under sterile conditions.

### 4.4 Rerouting towards the regulatory fate

The principal mechanism behind the clonal deletion of thymocytes bearing self-reactive TCRs involves the transduction of a robust TCR-mediated signal upon the recognition of tissue-restricted antigenic peptides, which requires the pro-apoptotic BH3-only Bim protein (112) and its increased association with the anti-apoptotic proteins Bcl-2 and Bcl-xL (113). Hence, the first requirement for the protection of thymocytes from negative selection-mediated elimination and redirection of their fate towards the Treg lineage is to restrain the activity of Bim (114). During inflammation, PRRs accomplish this by tweaking the thymocyte-intrinsic signalling network in multiple ways (**Figure 2**). First, TLRs repress Gfi1 in developing thymocytes (44), paving the way for their enhanced passage through the floodgates of β-selection as well as positive selection (115). This entails the possibility of progenitors receiving insufficient TCR signals (and potentially having self-reactive characteristics) to get permission to proceed along the T lymphopoietic pipeline, instead of falling prey to ‘death by neglect’. In fact, a recent study (116) has highlighted two distinct progenitor populations of thymic Treg cells: CD25+ and FoxP3low; which differ in terms of their developmental requirements, apoptotic tendency and TCR repertoire. Of these two, only the FoxP3low progenitors, which show remarkably low Nur77 expression (indicative of a weak TCR signal), rely on NF-κB activation for maturation. In parallel, thymocyte-intrinsic PRR stimulation activates c-Rel, an NF-κB family transcriptional regulator, which directly antagonizes Bim in thymocytes undergoing negative selection (117, 118). Inhibition of the c-Rel cascade by genetic ablation of TAK1 reveals this survival modulation to occur in an ERK-independent, JNK-dependent manner (119). Contrarily, activation of MDA5 due to the accumulation of unedited, adenosine-rich dsRNA in ADAR1-deficient thymocytes leads to their escape from negative selection and a resultant autoimmune response (120). These observations specifically accentuate the importance of PRRs in modulating the survival-death balance in relatively early T cell progenitors. Interestingly, dysregulated dsRNA sensing due to ADAR1-mediated RNA editing has even been causally linked to T cell leukaemia relapse (121). Besides, leukemic blasts arising from immature T cell progenitors exhibit particular enrichment of pattern recognition signalling-associated transcripts (122). Since the leukemic blasts carry pronounced signs of autoreactivity (123), the dysregulation of survival-death balance in autoreactive T cell progenitors by the PRRs may be associated with the induction of leukemic properties in them, providing them with a detour from clonal apoptosis. Of note, age or inflammation-associated thymic involution itself has been hypothesized to act as a deterrent to the emergence of leukemic clones (124). Therefore, more research is required to delineate the precise molecular checkpoints which govern a thymocyte’s potential leukemic conversion in response to PRR-mediated signals.

The story, nevertheless, does not end here. Thymi from c-Rel-/- mice have unchanged thymic cellularity as well as distribution of different thymocyte subsets, compared to their wild-type littermates (125). Even so, there is a noteworthy loss in terms of number as well as functionality of thymic Treg (tTreg) cells in the c-Rel-/- mice (126), which is not rescued even after transgenic overexpression of Bcl-2 to overcome the probable apoptotic manifestations. This indicates an additional cell-intrinsic function of c-Rel, beyond the restriction of Bim activity, in engineering their deviation towards the Treg fate. Incidentally, c-Rel directly binds the FoxP3 promoter through two distinct sites: -382 to -376 and -327 to -321; the same sites where NFAT binds (127). Moreover, c-Rel induces the formation of an ‘enhanceosome’ with four other transcription factors (p65, NFAT, CREB and Smad), where the latter two are recruited onto the distal enhancers of FoxP3 and scan along to the promoter upon enhanceosome formation (128). In addition, many of these pattern-dependent regulatory arms of tTreg development rely on altered proteostasis. While LUBAC-dependent linear ubiquitination and subsequent activation of NF-κB transcriptional activity are indispensable for tTreg generation (129), low-dose LPS accelerates the proteasomal degradation of FoxP3 in a Myd88-dependent manner (130). Baseline NLRP3 signalling also appears to disfavour tTreg generation (131). Furthermore, a continuous tussle between TLR signalling and FoxP3 shapes the metabolic fate of Treg cells. While the TLRs enforce glycolytic utilization of glucose via the PI3K-Akt-mTOR axis to increase Treg proliferation at the expense of their suppressive capacity, FoxP3 subverts these effects towards oxidative catabolism to restore their suppressive nature (132). Therefore, the PRR-FoxP3 axis seems to have a multi-layered regulation, involving several feedback loops which keep the tolerogenic outcomes under control (**Figure 2**).

## 5 Contextual impact of pattern recognition cascades on thymopoiesis: an evo-devo design?

Although the mechanisms responsible for the hormetic regulation of thymic function by innate immune sensing are conceivable, the evolutionary requirement for their selection and retention remains obscure. Hormesis is an adaptive strategy against stress, where cells/organisms benefit from the stress signal up to a threshold, beyond which it becomes detrimental (133). Even if one considers the molecular patterns as potential stress, why might the thymus benefit from biphasic kinetics of their impact on its activity?

The answer lies in the physiology of thymic function. Thymopoiesis and its quality control measures are intended to fend off foreign objects without affecting the host. During foetal life, the initial burst of T lymphopoietic events is targeted to generate a pool of T cells which do not break the immunologic tolerance at the maternal-foetal interface (134). On the other hand, tolerance needs to be established towards the freshly colonized commensal microorganisms right after the organism is born (135). Hence, the thymus needs a “two-component” system that enables the recognition of microbial products as well as maternally-derived danger signals, and kick-starts the T cell developmental proceedings; and at the same time, translates those signals into potential death-inducing messages when their intensities surpass a threshold. This ensures the efficient removal of thymocyte clones which can evoke a strong response against maternal antigens (for foetus) or commensal microbes (for neonates) to avoid potential autoimmunity; while clones that can elicit a moderate-intensity signal and potentially prevent trans-placental infections (for foetus) or opportunistic infections (for neonates), are selected. This continues in adulthood as well, where the sensitivity of developing thymocytes to the PRR-induced signals lets the system delete any thymocyte clone with strong ability of bystander activation, which can potentially turn autoreactive even without TCR-dependent signals. In parallel, an inflammatory dose-dependent control over thymic antigen presentation is also necessary, since tolerogenic selection mandatorily requires an optimal level of antigen presentation as per the principles of stochasticity. The thymus, therefore, has evolved to rely on the innate immune sensing cascades on a parsimonious mode, which not only acts proportionately with the extent of inflammation but can also function as a toggle between cell death and differentiation according to the signal intensity. This, however, must be restricted in certain stress conditions, where the system is infested by “altered self”. Examples include viral thymic infections, where the TECs display viral antigens and tolerize the thymocytes towards them (136). Infection-associated expedition of PRR-mediated TEC apoptosis takes care of this by poising the thymic architecture towards involution. A rise in the systemic load of microbial patterns with aging also serves the same function and prevents the thymus from producing autoreactive clones by molecular mimicry (137).

## 6 Challenges and perspectives

As discussed, pattern recognition is an easily tunable yet precisive switch for central tolerance. Following the perinatal microbial exposure, MAMP-dependent expansion of the thymic hematopoietic and stromal compartments prepares the ground for the upcoming TCR:pMHC encounters. Afterwards, PRRs multimodally promote the flux of thymocytes across the selection floodgates, creating a pool of self MHC-restricted T cells with defined antigenic specificities. While different PRRs apparently increase the yield of autoreactive thymocytes, sophisticated mechanisms dictate their diversion towards the Treg lineage to prevent autoimmune consequences. On the other hand, unique branches of PRR signalling activate death responses in the thymus and compromise tolerance induction during heightened, pathogenic inflammation; presumably to reduce the chances of molecular mimicry (69) and trigger thymic regeneration (73) in parallel. The most noteworthy point here is the contextuality of the PRR-mediated manifestations on thymic tolerance; where their tolerogenic functions under homeostatic conditions stand in complete contrast to their active consonance with the sustenance of autoreactivity in developing thymocytes. These autoreactive thymocytes, again in numerous pattern-dependent ways, are contextually diverted towards the Treg lineage or maintained as self-reactive conventional CD4+ and CD8+ T cell clones to act as sentinels to pathogenic invasion at the periphery. Thus, while silencing these PRR cascades to counter aging or infections may revert the thymic deterioration and apparently strengthen the immune defence, it may be accompanied by deleterious consequences in the long run.

The signalling machinery downstream of PRRs has heavy traffic to and from multiple other signalling pathways, forming a tightly woven signalling mesh (138, 139). Therefore, the ultimate tolerogenic outcome of the patterns largely depends on these auxiliary factors. As a result, their dynamic interplay with the intrathymic pattern signalling events may be manipulated to maintain thymic tolerance during PRR-directed therapeutic approaches. Interferons are integral executioners of PRR-mediated responses, where their multi-wave kinetics define their phenotypic manifestations on thymopoiesis and thymic tolerance (140–142). Hormones such as glucocorticoids (143, 144), leptin (145) etc differentially influence the thymic tolerogenic state in response to different patterns. These soluble mediators translate their actions inside the cell by modulating the levels of different intracellular messengers like calcium, reactive oxygen species (ROS) etc, which themselves have pronounced effects on central tolerance (146–149). The gut commensals play a pivotal part in regulating thymic tolerance by controlling the nature and levels of different MAMPs in the system (88, 150). Network-based approaches to unify the kinetics of all these factors in the thymic tolerogenic context may facilitate the application of immunosuppressive therapies with requisite modifications, which might not largely affect thymic tolerance while attenuating peripheral autoinflammation. Since different PRRs have overlapping yet markedly distinct effects on different tolerogenic checkpoints inside the thymus, contextual targeting of specific PRR signalling branches by focused approaches like targeted proteolysis (151, 152), rather than broad, pan-PRR-targeted approaches, may also be profitable for the preservation of thymic selection efficiency. In conditions of extensive, pathologic pattern-mediated thymic degeneration, bioengineered thymus organoids which can select the thymocyte clones with the desired specificities and affinities (153) may be implanted to maintain central tolerance. In addition, computationally designed peptides may be administered for being presented exclusively in the thymus, which can bind the altered TCR repertoire with optimal strength and select the requisite thymocyte clones at the expense of the rest (154). Controlled, thymus-specific activation of specific branches of PRR signalling may as well be used to foster thymic regeneration following inflammation-associated thymic injury (155).

## 7 Concluding remarks

The rising incidence of autoimmunity is a major concern for global healthcare, especially at the wake of virulent pandemics. Although heightened inflammation lies at the core of autoimmune pathogenesis, it is not clearly understood if generalized immunosuppression itself worsens the disease by affecting the intrathymic tolerogenic checkpoints. This review, as a whole, posits the generally vilified pattern recognition cascades as instrumental in maintaining thymic self-tolerance and discusses the mechanistic basis for that. The conclusions potentially unfold a new angle in the context of autoimmunity and its relation to inflammation, instigating surges of therapeutic repurposing against inflammatory pathologies to ensure smooth running of the tolerogenic machinery inside the thymus. It may be noted that the specific involvement of the dysfunctionalities of these PRR cascades in the context of human autoimmune diseases is relatively underexplored. Although a collapsed thymic tolerance has frequently been associated with several of these diseases (156, 157), understanding the involvement of PRRs in this phenomenon might unfold new avenues for the management of these diseases.
